# Quantitative Evaluation of Nanosecond Pulsed Laser-Induced Photomodification of Plasmonic Gold Nanoparticles

**DOI:** 10.1038/s41598-017-16052-7

**Published:** 2017-11-16

**Authors:** Andrew M. Fales, William C. Vogt, T. Joshua Pfefer, Ilko K. Ilev

**Affiliations:** Division of Biomedical Physics, Office of Science and Engineering Laboratories, Center for Devices and Radiological Health, U.S. Food and Drug Administration, 10903 New Hampshire Ave, Building 62, Silver Spring, MD 20993 USA

## Abstract

The rapid growth of gold nanoparticle applications in laser therapeutics and diagnostics has brought about the need for establishing innovative standardized test methods for evaluation of safety and performance of these technologies and related medical products. Furthermore, given the incomplete and inconsistent data on nanoparticle photomodification thresholds provided in the literature, further elucidation of processes that impact the safety and effectiveness of laser-nanoparticle combination products is warranted. Therefore, we present a proof-of-concept study on an analytical experimental test methodology including three approaches (transmission electron microscopy, dynamic light scattering, and spectrophotometry) for experimental evaluation of damage thresholds in nanosecond pulsed laser-irradiated gold nanospheres, and compared our results with a theoretical model and prior studies. This thorough evaluation of damage threshold was performed based on irradiation with a 532 nm nanosecond-pulsed laser over a range of nanoparticle diameters from 20 to 100 nm. Experimentally determined damage thresholds were compared to a theoretical heat transfer model of pulsed laser-irradiated nanoparticles and found to be in reasonably good agreement, although some significant discrepancies with prior experimental studies were found. This study and resultant dataset represent an important foundation for developing a standardized test methodology for determination of laser-induced nanoparticle damage thresholds.

## Introduction

Biophotonic application of plasmonic gold nanoparticles has become a highly active field of research in recent years due to their unique chemical and physical properties, such as high absorption cross sections and spectral tunability^1^. Many of the unique properties of gold nanoparticles are governed by the surface plasmon resonance (SPR) effect, a collective oscillation of electrons on the nanoparticle surface that occurs when excited with light at an appropriate wavelength. The SPR results in a strongly enhanced electromagnetic field near the particle surface, which causes unique, shape- and material-dependent spectral variations in light absorption and scattering. These properties allow nanoparticles to be used not only as therapeutic agents^2–4^, but also for diagnostic imaging^5,6^. The therapeutic effects produced by laser-nanoparticle interaction can occur through a variety of mechanisms. Photothermal transduction causes rapid heating in a localized area around the irradiated nanoparticles and has been used for the treatment of solid tumors *in vivo*, with both continuous-wave and nanosecond-pulsed lasers^7–9^. Photomechanical effects, such as cavitation, can occur when exposing plasmonic nanoparticles to pulsed laser light (nanosecond-to-femtosecond), and the resultant bubbles are capable of disrupting cancer cell membranes^10–12^. Photochemical effects, like the production of reactive oxygen species, have also been observed from laser irradiation of gold nanoparticles, with pulsed lasers having a greater effect than continuous-wave lasers^13,14^. The properties that make gold nanoparticles so effective for laser-based therapeutics can also lead to unintended side-effects in diagnostic procedures.

One of the most promising diagnostic techniques that may employ gold nanoparticle contrast agents is photoacoustic imaging (PAI), a rapidly maturing biomedical modality capable of macro- and micro-scale imaging^5^. In PAI, tissue is illuminated with a nanosecond pulsed laser, typically a Q-switched Nd:YAG with pulse duration in the range of 5–10 ns, at exposures below standard safety limits^15^. This exposure causes rapid optical absorption and thermal expansion that produces acoustic waves, which can be detected using an ultrasonic transducer. The use of gold nanoparticles as contrast agents for PAI has the potential to generate photothermal, photomechanical, or photochemical effects that result in tissue injury. Furthermore, the spectral changes brought about by nanoparticle photomodification can cause spectral shifts in absorption that degrade PAI-nanoparticle product performance^16^.

While there are well-established laser safety standards for the skin and eye, these standards are insufficient when exogenous chromophores such as nanoparticles are present. Additionally, no standards exist for evaluating the performance of laser-nanoparticle combination products for medicine. In recent years, a number of studies have contributed knowledge on the laser-nanoparticle interaction processes and resultant bioeffects, as well as test methods that could form the basis of new standards. One key step in elucidating laser-nanoparticle interactions is to understand the photostability of nanoparticles and characterize laser induced photomodification processes, including photothermally-induced melting/reshaping effects that have been documented for different types of metallic nanoparticles^16–22^. We chose to study spherical gold nanoparticles as their surface plasmon resonance is close to the wavelength (532 nm) of the second-harmonic Q-switched Nd:YAG laser, which is broadly employed in biophotonics and photomedicine^23–25^, and because these nanoparticles possess well-defined morphological properties and their spherical shape is well-suited for theoretical investigation.

Several prior studies have evaluated the effect of pulsed laser irradiation on gold nanospheres – more accurately gold “pseudospheres” since most of the particles studied, as well as commercially available versions, have faceted rather than smooth edges. One such study by Takami *et al*. looked at the size reduction of approximately 50 nm gold nanospheres at different pulse energies^18^. Werner *et al*. investigated the effect of irradiation wavelength on 55 nm gold nanospheres and was mostly concerned with modeling the difference between interband and intraband excitation^22^. Another study used dynamic light scattering (DLS), electron microscopy and UV-Vis spectroscopy to examine the impact of a single laser pulse on particles of different sizes^26^. While results were presented for a range of energy levels, specific damage thresholds were not determined and comparison with theoretical results was not presented. These prior studies have provided significant insights, yet are deficient in terms of generating a standardized test methodology as they either only used a single nanoparticle size or provided a qualitative description of the damage with no well-defined threshold.

Our overall objective was to facilitate the optimization of safety and effectiveness in emerging biophotonic products incorporating nanoparticles. Previous works have not established a common approach for assessing laser damage thresholds in nanoparticles, and information on the role of particle size in nanosphere damage is typically spread across multiple reports with inconsistent experimental conditions. Therefore, the goals of this proof-of-concept study were to implement and assess methodologies to quantitatively determine nanoparticle photomodification thresholds, and to generate data on the melting process and damage thresholds that can be used to improve understanding of this process. Specifically, we have conducted quantitative experimental and analytical investigation of the interaction between nanosecond laser pulses at 532 nm and plasmonic gold nanoparticles with diameters from 20 to 100 nm over a wide range of laser radiant exposures.

## Experimental

### Materials and Methods

**Figure 1:**
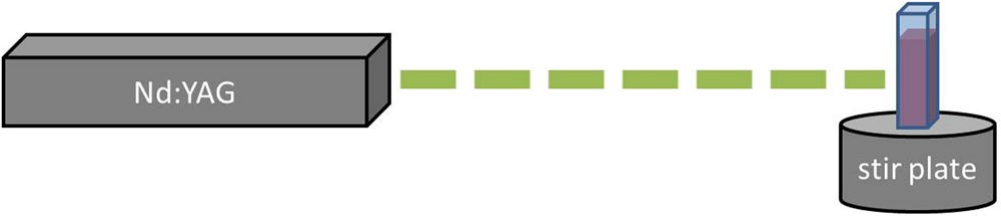
Schematic representation of the experimental setup.

Gold nanoparticle samples of nominal diameter size 20 nm, 40 nm, 60 nm, 80 nm, and 100 nm were purchased from Cytodiagnostics (Burlington, Ontario, Canada). The nanoparticle solutions, specified to be >95% spherical, were supplied in citrate buffer (0.1 mg/mL) at 1 OD and used as received. A frequency-doubled, Q-switched Nd:YAG laser (Surelite I-10 with SSP-2, Continuum; Santa Clara, CA) was used to provide 532-nm-wavelength 5-ns-duration pulses at a 10 Hz repetition rate. The laser pulse energy was controlled using a Glan-Taylor polarizer (GL10, Thorlabs; Newton, NJ) placed at the laser aperture. Radiant exposures from 0 up to 150 mJ/cm^2^ were used depending on particle size. For each nanoparticle exposure, the stock nanoparticle solution (3 mL) was placed into a disposable plastic cuvette (#9002, Perfector Scientific, Inc; Atascadero, CA) with a magnetic stir bar (Z363545, Sigma-Aldrich; St. Louis, MO) under rapid mixing (1000 rpm). Unfocused laser beam (3.5 mm spot size, Gaussian beam profile) was delivered perpendicular to one face of the cuvette for 5 minutes to ensure a homogenous exposure of the sample. Laser irradiation was carried out in triplicate for each value of radiant exposure and particle size. A schematic of the experimental setup is shown in Fig. 1.

Laser-exposed samples were prepared for TEM analysis by drop-casting particle solution (7 µL) onto a formvar/carbon coated copper grid (FCF400-Cu; Electron Microscopy Sciences, Hatfield, PA) and allowing the droplet to evaporate overnight. Particle diameter measurements of the TEM images were performed using ImageJ 1.51k (NIH; Bethesda, MD) by fitting an ellipse to each particle. The particle diameter was defined as the major axis length of the fitted ellipse. At least 50 particles were measured for each particle size and radiant exposure to determine the average diameter and standard deviation. Data analysis was performed using NumPy and Matplotlib within the Enthought Canopy Python distribution^27,28^. Numerical simulations were performed using the Matlab code provided by Metwally *et al*. in the supporting information of their manuscript^29^. Discrete dipole approximation (ADDA 1.3b4) was used to calculate the extinction spectra of spherical and quasispherical particles^30^.

### Instrumentation

The laser beam spot size (1/e width) was measured using a beam profiler (Beamstar-FX-50; Ophir, Israel), while laser pulse energy was measured with a calibrated pyroelectric energy sensor (PE10-SH-V2; Ophir). Transmission electron microscopy (TEM) micrographs were taken on a JEM-1400 (JEOL Inc., Peabody, MA) operating at an accelerating voltage of 80 kV. Optical extinction spectra were collected over 400–800 nm using a dual-beam UV/VIS/NIR spectrophotometer (Lambda 1050; PerkinElmer, Waltham, MA) with 10 mm path length plastic cuvettes. Dynamic light scattering (DLS) analysis was performed with a Zetasizer Nano ZS (Malvern, Worcestershire, UK).

### Data Availability Statement

The datasets generated during and/or analyzed during the current study are available from the corresponding author on reasonable request.

## Results and Discussion

### Experimental Characterization of Pulsed Laser-Nanoparticle Interaction

**Figure 2:**
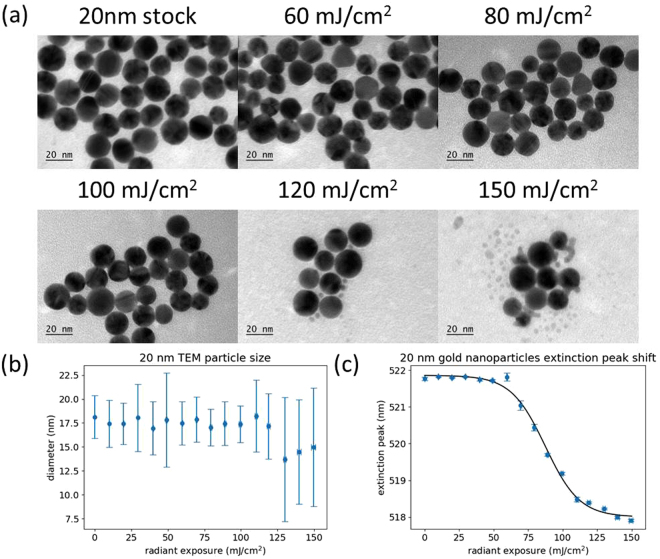
(**a**) Representative TEM images of 20 nm gold nanoparticles after different radiant exposures. (**b**) Particle size measurements calculated from TEM images. (**c**) Plasmon peak wavelength of the nanoparticle sample after different radiant exposures, determined by spectrophotometry. Error bars are ±1 standard deviation.

Figure 2 presents the characterization of 20 nm gold nanoparticles after laser exposure over a range of radiant exposures. As shown in Fig. 2(a), the original, faceted, quasispherical particles are partially reshaped into highly spherical particles at a radiant exposure of 80 mJ/cm^2^, with a majority of particles becoming smooth spheres after irradiation at 100 mJ/cm^2^. At higher radiant exposures, small particle fragments began to appear, accompanied by a decrease in mean particle size relative to the stock nanoparticles. The reshaping and size reduction effects observed here have been reported extensively in the literature and can be attributed to nanoparticle melting and surface evaporation, respectively^20,22,31^. Figure 2(b) shows that there was little change in the mean particle size as radiant exposure was increased, with a slight increase in standard deviation above 120 mJ/cm^2^. This increased variation may be due to the presence of particle fragments as well as particles of reduced size due to evaporation. Once particle evaporation occurs, irregular and elongated particle fragments began to appear, reducing the average roundness of the sample and increasing the variability. Figure 2(c) shows plasmon peak wavelength determined by spectral extinction measurements as a function of laser radiant exposure. Interestingly, the plasmon peak wavelength is highly sensitive to the reshaping of the particle surface from faceted to smooth. There is a sharp decrease in plasmon peak wavelength as the particles melt, followed by a plateau where there is a gradual decrease in the peak wavelength as the particles evaporate and form smaller fragments. For these 20 nm particles, reshaping occurs over a wide range of radiant exposures (spanning approximately 50 mJ/cm^2^). Representative extinction spectra of the 20 nm gold particles at different radiant exposures are shown in Supplementary Fig. S1.

**Figure 3:**
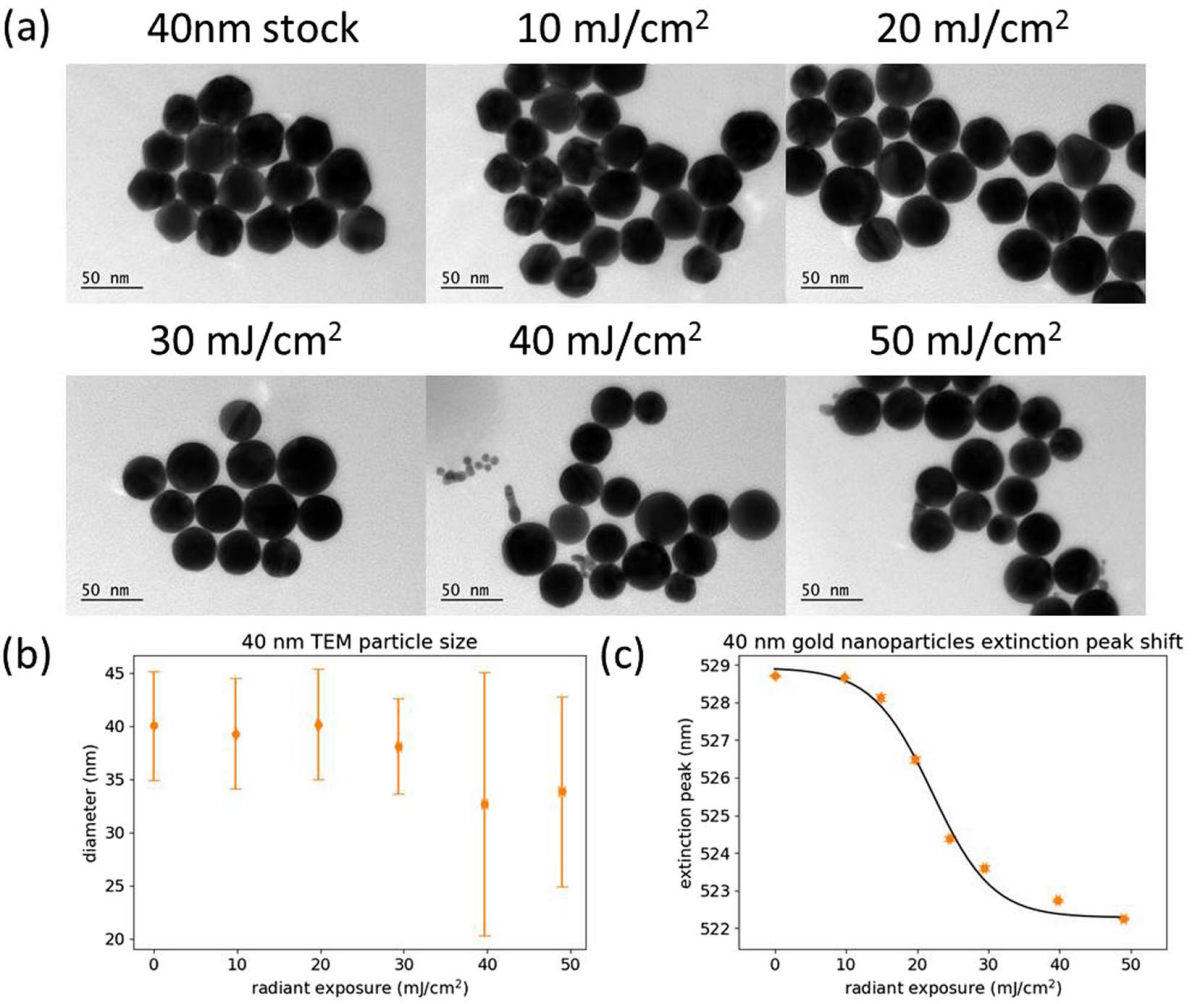
(**a**) Representative TEM images of 40 nm gold nanoparticles after different radiant exposures. (**b**) Particle size measurements calculated from TEM images. (**c**) Plasmon peak wavelength of the nanoparticle sample after different radiant exposures, determined by spectrophotometry. Error bars are ±1 standard deviation.

The characterization of 40 nm gold nanoparticles after laser irradiation is shown in Fig. 3. From Fig. 3(a), it can be seen that the 40 nm particles began to reshape at a radiant exposure of around 20 mJ/cm^2^, with nearly all of the particles converted into smooth spheres by 30 mJ/cm^2^. At 40 mJ/cm^2^ and above, we began to observe particle evaporation and size reduction. This dramatic reduction in the radiant exposure required for photomodification is a result of less efficient heat diffusion to the surrounding medium^29,32^. Quantitative analysis of TEM images agrees well with qualitative observations associated with Fig. 3(b). As the particles melt and reshape, the mean size does not change significantly but the aspect ratio becomes more uniform, resulting in a slight decrease in the standard deviation. The onset of particle evaporation coincides with a decrease in particle size, along with an increase in measured size standard deviation. Figure 3(c) shows the same trend in plasmon peak shift for 40 nm particles that was observed with 20 nm particles. Correlation of the curve with TEM images again indicates that the sharp initial decrease in plasmon peak position is related to the melting and reshaping of the nanoparticles, whereas particle evaporation and size reduction displays a more gradual shift in the peak. Here, the melting and reshaping has been completed within approximately 20 mJ/cm^2^ of the radiant exposure where it began. Representative extinction spectra at different radiant exposures can be found in Supplementary Fig. S2.

**Figure 4:**
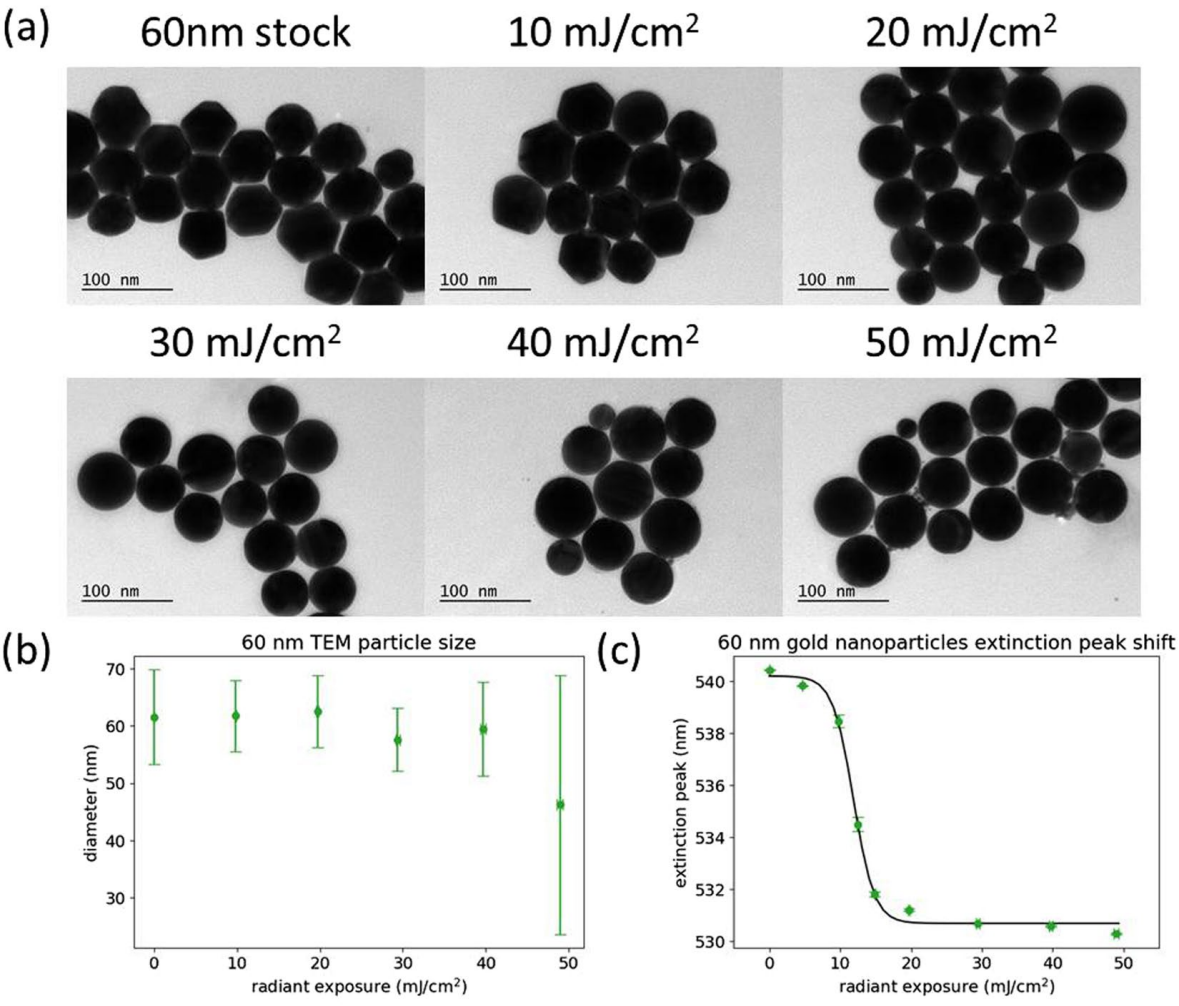
(**a**) Representative TEM images of 60 nm gold nanoparticles after different radiant exposures. (**b**) Particle size measurements calculated from TEM images. (**c**) Plasmon peak wavelength of the nanoparticle sample after different radiant exposures, determined by spectrophotometry. Error bars are ±1 standard deviation.

Figure 4 shows the characterization of 60 nm gold nanoparticles that have been irradiated at different levels of radiant exposure. The TEM images (Fig. 4(a)) show particle melting to be completed by 20 mJ/cm^2^, followed by evaporation and size reduction becoming apparent at 40 mJ/cm^2^. The measured particle sizes (Fig. 4(b)) showed a decrease in particle size starting at 30 mJ/cm^2^ and an increase in the standard deviation. Figure 4(c) displays the response of the plasmon peak position, having a steep drop until particle melting was complete, and then a slowly decreasing plateau as particle evaporation occurred, as is to be expected from the previous results for 20 and 40 nm particles. The 60 nm particles have a much narrower window of radiant exposure for melting to occur (around 10 mJ/cm^2^) compared to the smaller sized particles. Supplementary Fig. S3 shows a representative sample of the extinction spectra that were used to create Fig. 4(c).

**Figure 5:**
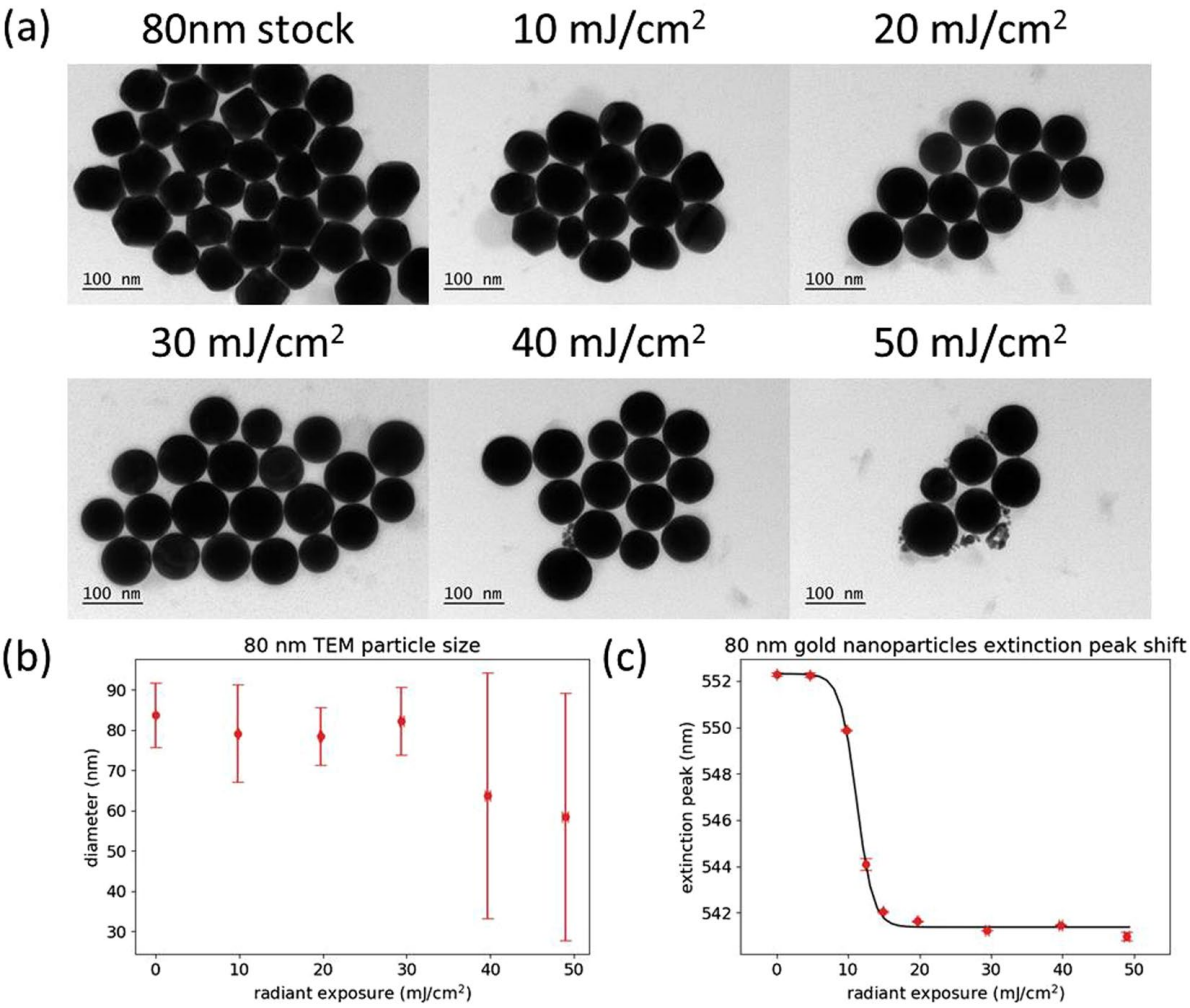
(**a**) Representative TEM images of 80 nm gold nanoparticles after different radiant exposures. (**b**) Particle size measurements calculated from TEM images. (**c**) Plasmon peak wavelength of the nanoparticle sample after different radiant exposures, determined by spectrophotometry. Error bars are ±1 standard deviation.

Similar results were obtained using 80 nm gold nanoparticles (Fig. 5). At 10 mJ/cm^2^ there was already a significant fraction of smooth particles observed by TEM. Complete melting and reshaping had occurred by 20 mJ/cm^2^, while evaporation and size reduction was not seen until 40 mJ/cm^2^. The measured size of the particles remained constant up to 30 mJ/cm^2^, after which small fragments began to appear, reducing the average particle size and increasing the standard deviation. The range of radiant exposure between onset and completion of melting appears to be slightly lower than that observed for the 60 nm particles (less than 10 mJ/cm^2^). Representative extinction spectra of the 80 nm particles are shown in Supplementary Fig. S4.

**Figure 6:**
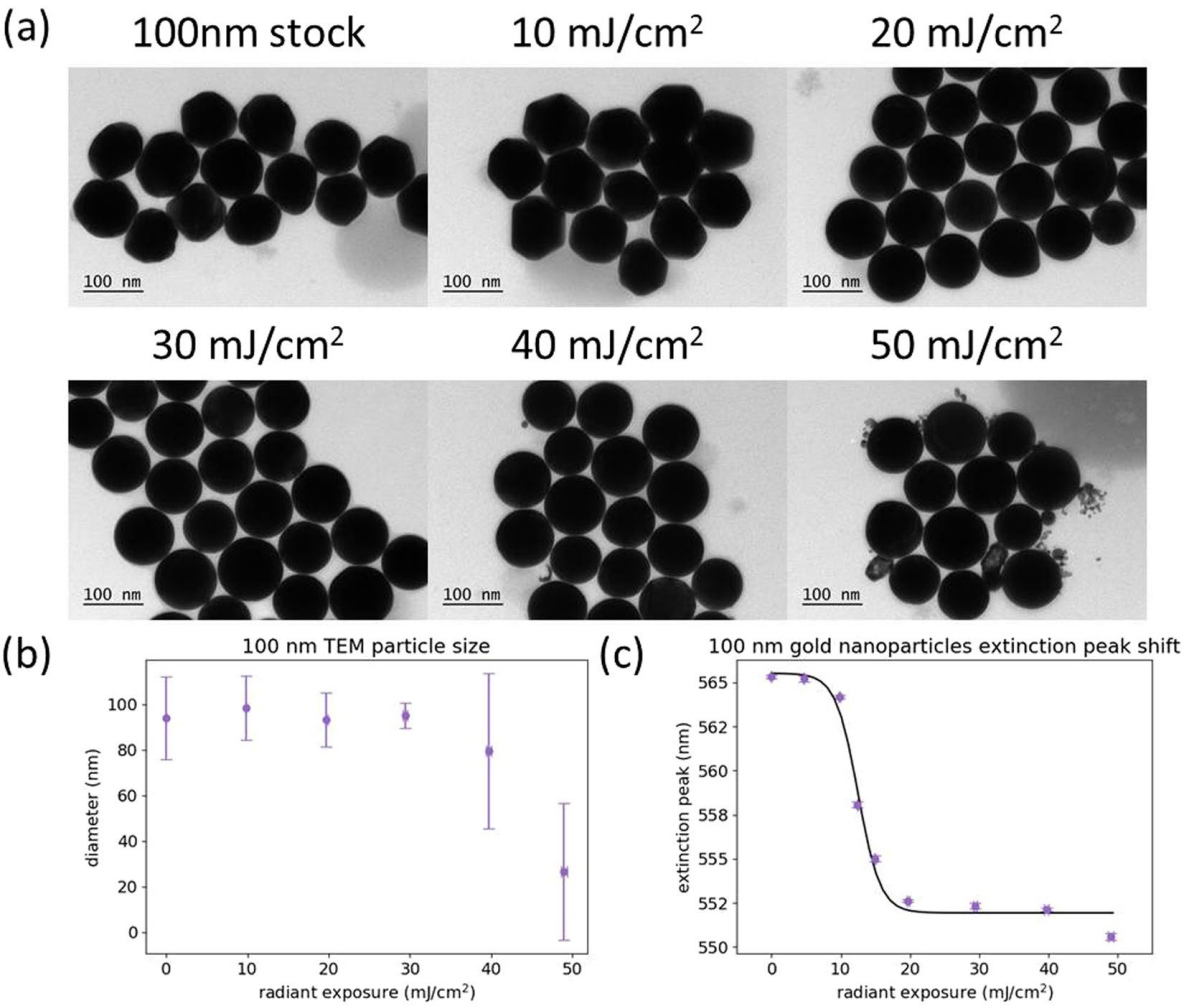
(**a**) Representative TEM images of 100 nm gold nanoparticles after different radiant exposures. (**b**) Particle size measurements calculated from TEM images. (**c**) Plasmon peak wavelength of the nanoparticle sample after different radiant exposures, determined by spectrophotometry. Error bars are ±1 standard deviation.

The 100 nm particles had a slightly higher threshold for melting than the 80 nm particles. At a radiant exposure of 10 mJ/cm^2^, there were no smooth particles observed by TEM, while most particles had been reshaped at 20 mJ/cm^2^ (Fig. 6(a)). Reshaping is completed by 30 mJ/cm^2^, which can also be observed from the measurements of particle size (Fig. 6(b)). At 40 mJ/cm^2^ and above, the presence of small fragments caused a decrease in average particle size, along with an increase in the standard deviation. The onset and completion of melting occurred over a range of radiant exposure that is slightly above 10 mJ/cm^2^, which is higher than that of both the 60 nm and 80 nm particles, but lower than that of the 20 nm and 40 nm particles. Representative extinction spectra are presented in Supplementary Fig. S5.

**Figure 7:**
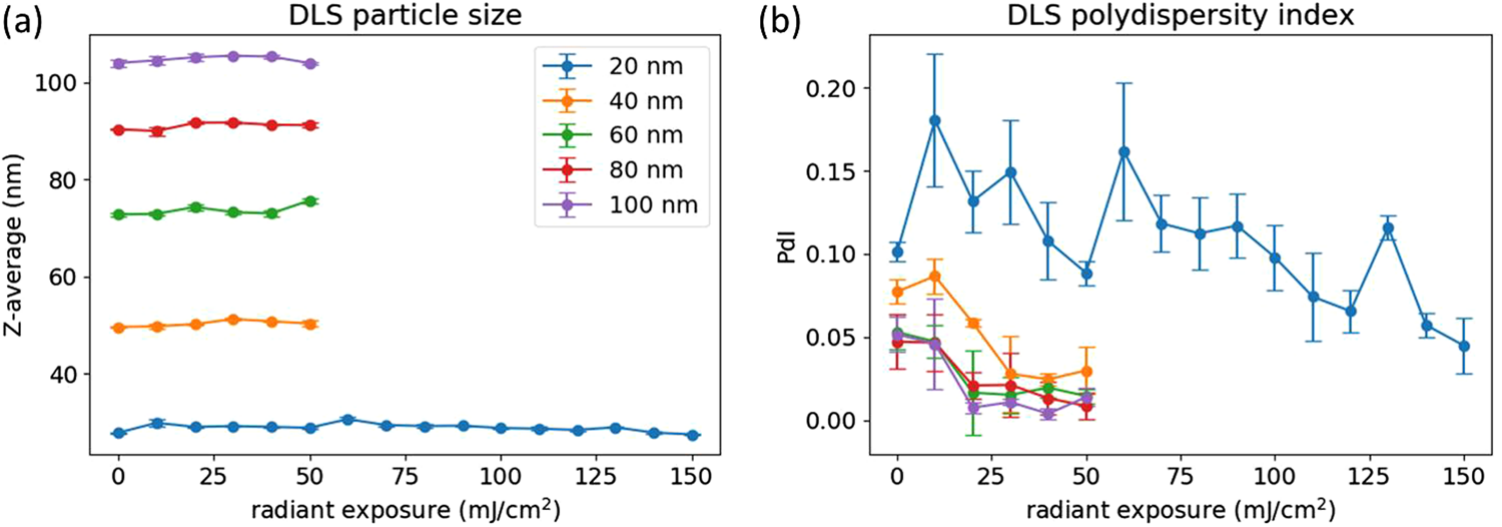
DLS measurements of particle size (**a**) and polydispersity index (**c**) for the 5 sizes of nanoparticles at different radiant exposures.

Dynamic light scattering (DLS) was another tool used to characterize the nanoparticle samples after laser exposure. As shown in Fig. 7(a), there was little to no change observed in particle size for any of the particles or radiant exposures used. As noted in the previously shown TEM images, the overall particle size did not change much until small fragments were generated due to evaporation. Since the scattering intensity scales with particle radius, r^6^, the DLS-measured particle size will be skewed toward any larger particles in the sample. The polydispersity index (PdI), a measurement of the heterogeneity of a sample, is another parameter that is important for nanoparticle characterization. From our experimental data, all 5 of the nanoparticle samples show the same trend of decreasing PdI with an increase in radiant exposure (Fig. 7(b)). This decrease was to be expected after considering the particle reshaping observed by TEM. The original samples contained quasispherical, faceted particles of various shapes. After reshaping, all of the original particles have become spherical, resulting in a more uniform diffusion coefficient throughout the sample, and thus a reduction in the PdI. While DLS is a commonly used technique for the characterization of nanoparticle samples, the inherently low resolution precludes its use in analyzing the photodegradation of gold nanoparticles under pulsed-laser irradiation.

After comparing the three different characterization techniques, spectrophotometry was found to be the most accurate, reliable and least burdensome method for assessing nanosphere photodegradation. The plasmon peak position as a function of radiant exposure was used to quantitatively determine the damage thresholds of each particle size studied by means of curve fitting. A four-parameter sigmoid regression was performed, with the 50% point defined as the damage threshold, similar to the determination of dose response curves used in pharmacological research as well as the tissue-damage thresholds defined in ANSI Z136.1 laser safety standard^15,33–35^. As needed, more conservative or extreme thresholds could be determined by choosing a different portion of the response curve (*e.g*., at 10% or 90% levels) due to the high sensitivity and low standard deviations of the measurements.

The abrupt change in plasmon peak wavelength that occurs after particle melting and reshaping can be explained by the reduction of multipolar resonances^36^. Elimination of the particle facets improves symmetry, increasing the efficiency of the dipolar resonance and blue-shifting the plasmon peak. This shifting has also been observed experimentally in the reshaping of octahedra into spheres, where a shift of over 30 nm is reported for 75 nm particles^37^. To illustrate the shape effect on the plasmon resonance peak, discrete dipole approximation was used to calculate the extinction spectra of a spherical and quasispherical model (Supplementary Fig. S6). It can be seen that transitioning from the quasispherical to spherical shape produces a significant blue-shift in the plasmon peak, as expected from our experimental results and previous studies found in the literature.

As described above, the plasmon peak shift during particle fragmentation is much less pronounced than what was observed during melting. If one were interested in determining fragmentation thresholds by spectrophotometry, a different metric would have to be used. It may be possible to use the change in absorbance at the plasmon peak to determine the fragmentation thresholds in a similar manner to the damage thresholds due to melting determined in this work. Previous reports have shown the change in absorbance to be relatively stable during the range of exposure where melting occurs, with a steeper drop-off once fragmentation begins^22,31^.

### Theoretical Investigation of Pulsed Laser-Induced Damage Thresholds

We have also employed theoretical modeling to study the size-dependent damage threshold of spherical gold nanoparticles. The optical properties of gold nanoparticles were calculated using Mie theory^38^. Supplementary Fig. S7 shows the calculated extinction spectra for gold nanospheres of 20, 40, 60, 80, and 100 nm diameters. The calculated spectra were then compared to the experimentally measured spectra of particle samples with the same nominal diameters (Supplementary Fig. S7). The slight deviations between theory and experiment can be attributed to several factors: the nanoparticles are not completely monodisperse, the actual mean size may differ from the nominal specified value, and the stock particles are faceted spheroids, which present different plasmon resonance effects than ideal spherical particles.

Calculation of damage thresholds was performed by adopting the numerical method used by Metwally *et al*. and adjusting the parameters to match our experimental conditions^29^. Particle absorption cross-sections were obtained from Mie theory. A pulse width of 5 ns and Kapitza resistivity of g = 150 MW m^−2^ K^−1^ were used in all simulations and the maximum temperature reached within the nanoparticle was recorded. The damage threshold was calculated in a similar manner to the bubble generation threshold calculated by Metwally *et al*.:

$${H}_{m}={H}_{0}\frac{{T}_{m}-{T}_{0}}{\delta {T}_{max}}$$

**Figure 8:**
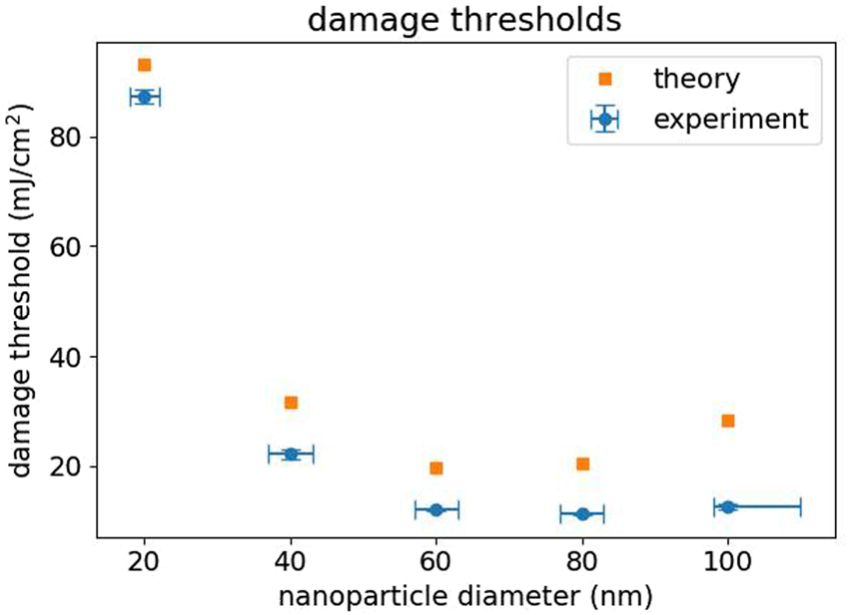
Comparison between the experimental (blue) and theoretical (orange) damage thresholds for gold nanospheres of different sizes. Error bars for the experimental data are ±1 standard deviation.

In this case, *H*_0_ is the incident radiant exposure (mJ/cm^2^), *T*_*m*_ is the melting temperature of gold (1337 K), *T*_0_ is the ambient temperature (293 K), and *δT*_*max*_ is the maximum temperature achieved within the nanoparticle. As shown in Fig. 8, the theoretically determined damage thresholds retain the same bathtub shape as a function of particle size that has been reported for bubble formation thresholds, with a minimum radiant exposure around 60 nm. This bathtub shape results from the fact that the absorption cross section is no longer proportional to the volume of the nanoparticle above a diameter of 60 nm^29^. The experimental damage thresholds also follow a bathtub shape, though with a minimum radiant exposure found for particles with 80 nm diameter. The theoretical and experimental damage thresholds are listed in Table 1 for ease of comparison, and calculated damage thresholds are in reasonably good agreement with the experimentally determined thresholds.

**Table 1:** Gold nanosphere damage thresholds tabulated from the data points in Fig. 8.

Nanosphere Diameter	Threshold (mJ/cm^2^)	
	theory	experiment
20 nm	93.1	87 ± 1
40 nm	31.7	22.1 ± 0.8
60 nm	19.7	11.9 ± 0.2
80 nm	20.5	11.2 ± 0.1
100 nm	28.3	12.5 ± 0.4

There are a number of factors that could be contributing to the observed differences between experimentally and theoretically determined nanoparticle damage thresholds. One factor, as previously discussed, is the heterogeneity of size and shape within a colloidal sample of nanoparticles. Another factor is that the effect of surface melting of nanoparticles, which can occur below the bulk melting point, was not considered in the theoretical model^20^. It has been suggested that this surface pre-melting originates from the vertices and edges of facets on the nanoparticle surface, where higher plasmon intensities could induce melting at lower thresholds than would be expected for truly spherical particles^39–41^. The model is also based on the interaction of a single laser pulse with an individual nanoparticle, while in our experiment, we delivered multiple pulses to ensure that the total sample volume was homogenously exposed to the laser. Although the pulse-to-pulse delay (∼100 ms) was much longer than the thermal relaxation time for gold nanoparticles (<1 ns), it is conceivable that some nanoparticles were exposed to multiple pulses and could have an increased probability of melting and reshaping in the radiant exposure range near the threshold. This would cause the experimentally determined thresholds to shift toward lower radiant exposures. Additionally, the single-particle model also does not account for the effect of nanoparticle scattering, which could lead to more of the laser energy being transferred to the particle solution than would be predicted from absorption alone, again lowering the experimentally determined thresholds, especially for the 80 and 100 nm particles where scattering contributes significantly to the overall extinction^42^.

### Comparison with Previous Works

Our experimental results provide a much more comprehensive characterization of the damage threshold for gold nanospheres using 532 nm nanosecond pulses than provided in the literature, yet are consistent with the limited data that have been published. For example, we measured a damage threshold of 11.9 ± 0.1 mJ/cm^2^ for 60 nm particles which agrees well with the experimentally determined value of ∼12.2 ± 2 mJ/cm^2^ observed by Werner *et al*. for 55 nm particles^22^. However, this study was limited in that it did not provide results for any other particle sizes. Werner *et al*. also used the change in absorbance at the plasmon peak to monitor particle melting, which appears to be less sensitive than using the plasmon peak position – at the first observed change in absorbance, all of the particles have already melted and reshaped into perfect spheres according to the TEM images presented.

In an additional comparison to a study presented by Cavicchi *et al*., since this work did not explicitly determine and state damage thresholds for the different particle sizes that were investigated, we have estimated damage thresholds from data presented in this study (Figure 4)^26^ for comparison with our results (Supplementary Fig. S8). The higher threshold levels found by Cavicchi *et al*. are likely due to the use of single pulses, and unfortunately, no error bars were provided for these data. These results also indicated a similar local minimum effect, with the lowest threshold found in the range of 60 nm particles. However, significant discrepancies were seen between the magnitude of this threshold change with particle size based on the wavelength shift compared to the peak absorbance or theoretical predictions. It is worth noting that the aforementioned Werner *et al*. results were determined from samples that had undergone an extended exposure consisting of multiple laser pulses, similar to our study, and the resulting threshold seems to fall along the same curve as thresholds determined from our measurements. The use of multi-pulse exposures provides the benefit of producing a more uniform sample by reducing the effects of pulse-to-pulse energy variation and beam inhomogeneity in addition for ensuring more clinically relevant application conditions. To verify that the reshaping and fragmentation effects observed in the representative TEM images are indicative of the whole sample, low magnification images with a larger number of particles are presented for the 40 nm diameter particles in Supplementary Fig. S9.

Another important consideration in the effects of laser-nanoparticle interaction is the laser pulse duration. Pulses in the 5–10 ns range, as were used in this study, are the most relevant for current biophotonic therapeutics and diagnostics. However, the use of shorter or longer pulses may be desired in certain cases. Previous work has shown that decreasing pulse duration lowers the threshold for damage in plasmonic nanoparticles^29,43^. In the case of the gold nanospheres used in this study, it is likely that the test method proposed here for damage threshold determination by spectrophotometry will remain valid regardless of the pulse duration used and any differences in threshold this may cause. Future work will evaluate the use of this test method for different shapes of plasmonic nanoparticles such as rods and shells.

## Conclusion

We have investigated the effects of a nanosecond pulsed laser on spherical gold nanoparticles, and identified suitably robust methods for detecting and characterizing laser-induced changes in particle morphology such as melting, reshaping, and fragmentation. We also demonstrated the applicability of this methodology for quantitative determination of particle damage thresholds as functions of particle diameter and radiant exposure. These results will aid in future development of standardized test methods for evaluating safety and effectiveness of emerging NP-based medical products.

## Electronic supplementary material

Supplementary Information
