# Author Correction: Quantitative Evaluation of Nanosecond Pulsed Laser-Induced Photomodification of Plasmonic Gold Nanoparticles

**DOI:** 10.1038/s41598-018-24794-1

**Published:** 2018-04-18

**Authors:** Andrew M. Fales, William C. Vogt, T. Joshua Pfefer, Ilko K. Ilev

**Affiliations:** Division of Biomedical Physics, Office of Science and Engineering Laboratories, Center for Devices and Radiological Health, U.S. Food and Drug Administration, 10903 New Hampshire Ave, Building 62, Silver Spring, MD 20993 USA

Correction to: *Scientific Reports* 10.1038/s41598-017-16052-7, published online 16 November 2017

The original version of this Article contained a typographical error in the spelling of the author T. Joshua Pfefer, which was incorrectly given as Joshua Pfefer.

Additionally, the Author Contributions section in this Article was incomplete.

“A.F. performed all experiments and simulations and wrote the initial draft of this manuscript. W.V., J.P. and I.I. provided guidance and feedback throughout the study. All authors edited and reviewed the manuscript.”

now reads:

“A.M.F. performed all experiments and simulations and wrote the initial draft of this manuscript. W.C.V., T.J.P. and I.K.I. provided guidance and feedback throughout the study. All authors edited and reviewed the manuscript. T.J.P and I.K.I conceived and planned the study.”

These errors have now been corrected in the PDF and HTML versions of the Article, and in the accompanying Supplementary Information.

