# Supplementary material for: Quantitative Evaluation of Nanosecond Pulsed Laser-Induced Photomodification of Plasmonic Gold Nanoparticles
Source: Sci Rep. 2017 Nov 16;7:15704. doi: 10.1038/s41598-017-16052-7 (PMC5691067; doi:10.1038/s41598-017-16052-7)
Supplement: Supplementary file 1 — Supplementary Information [file 41598_2017_16052_MOESM1_ESM.pdf]

Supplementary Information for:

Quantitative Evaluation of Nanosecond Pulsed Laser-Induced Photomodification of Plasmonic Gold Nanoparticles

Andrew M. Fales*, William C. Vogt, T. Joshua Pfefer, and Ilko K. Ilev

Office of Science and Engineering Laboratories, Center for Devices and Radiological Health
U.S. Food and Drug Administration
10903 New Hampshire Ave, Building 62, Silver Spring, MD 20993

**Corresponding author: andrew.fales@fda.hhs.gov*

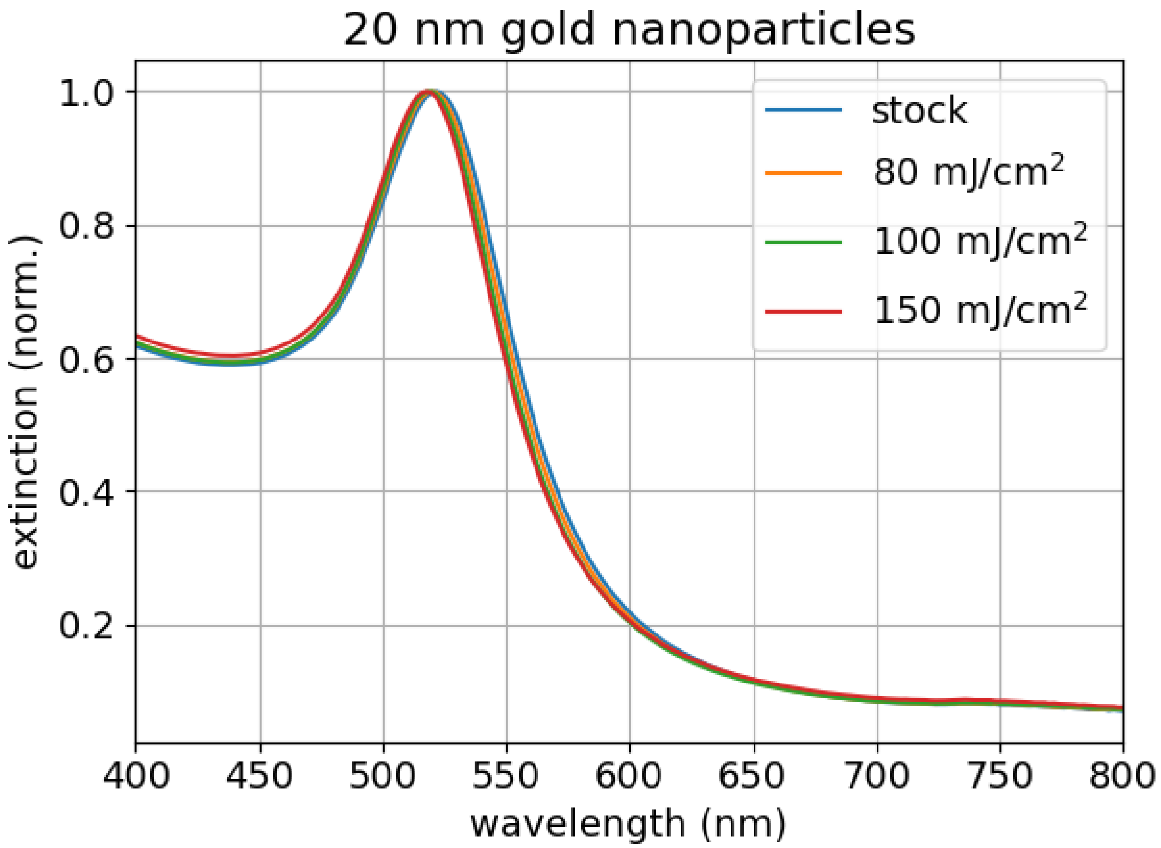

Figure S1. Representative extinction spectra of 20 nm gold nanoparticles after laser irradiation at the indicated radiant exposures.

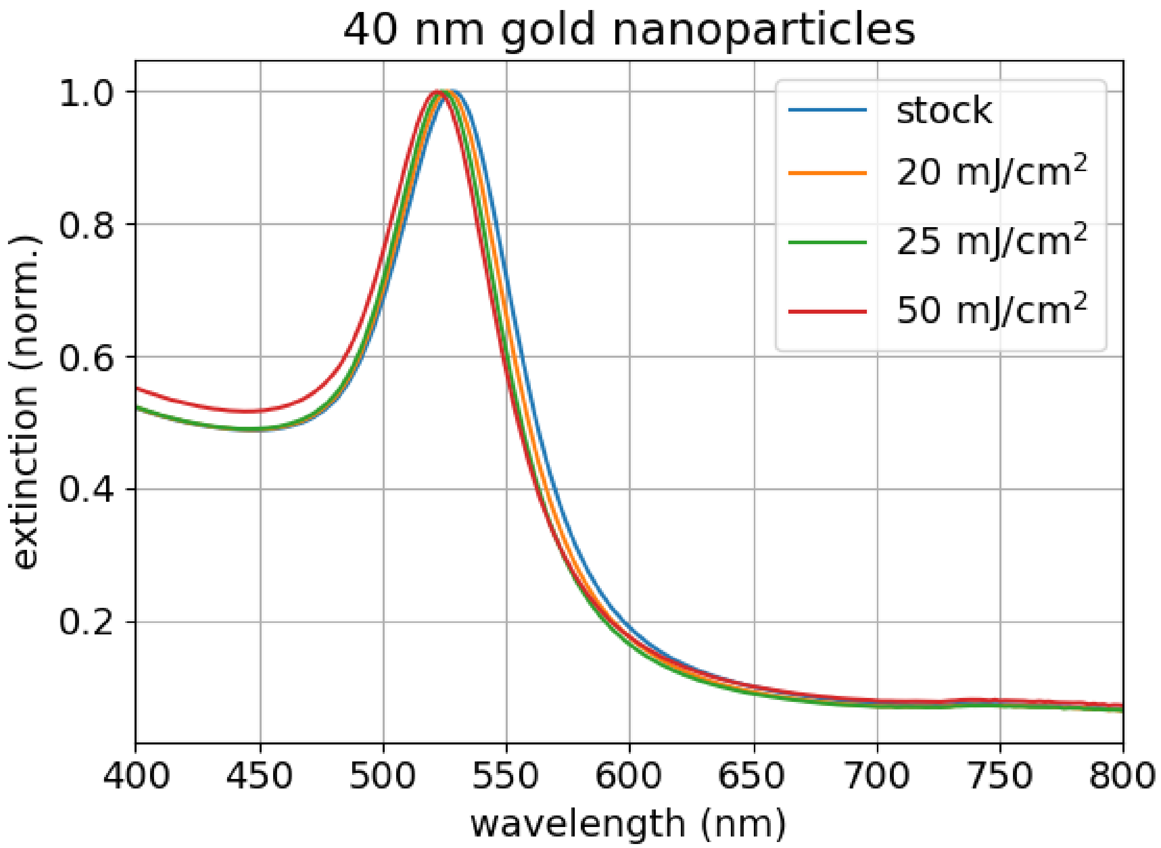

Figure S2. Representative extinction spectra of 40 nm gold nanoparticles after laser irradiation at the indicated radiant exposures.

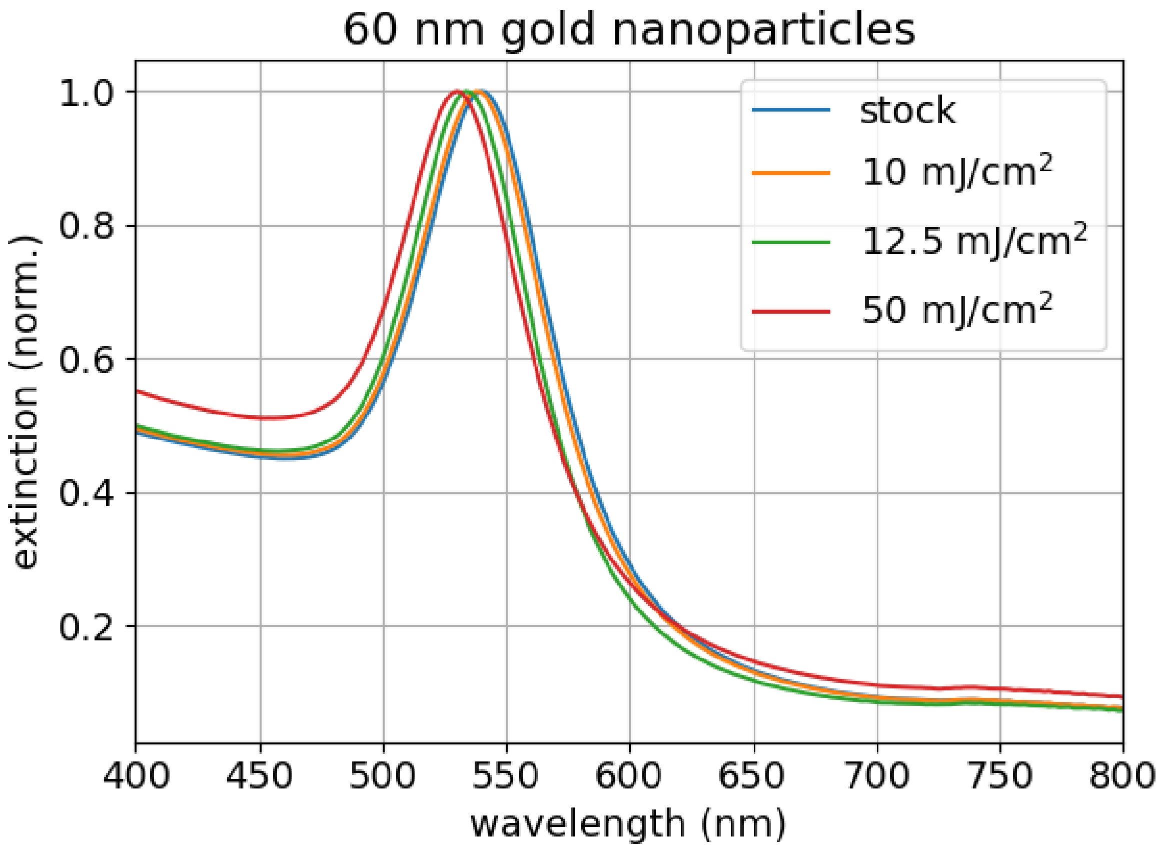

Figure S3. Representative extinction spectra of 60 nm gold nanoparticles after laser irradiation at the indicated radiant exposures.

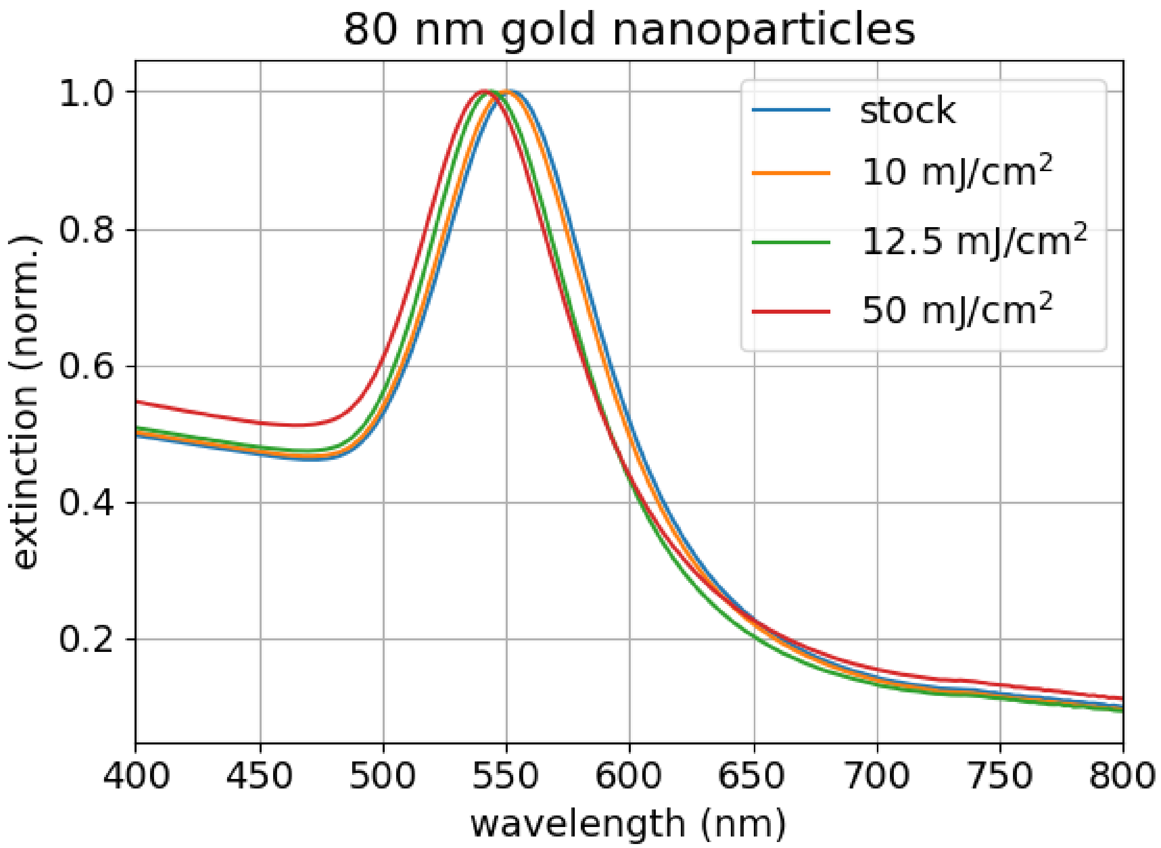

Figure S4. Representative extinction spectra of 80 nm gold nanoparticles after laser irradiation at the indicated radiant exposures.

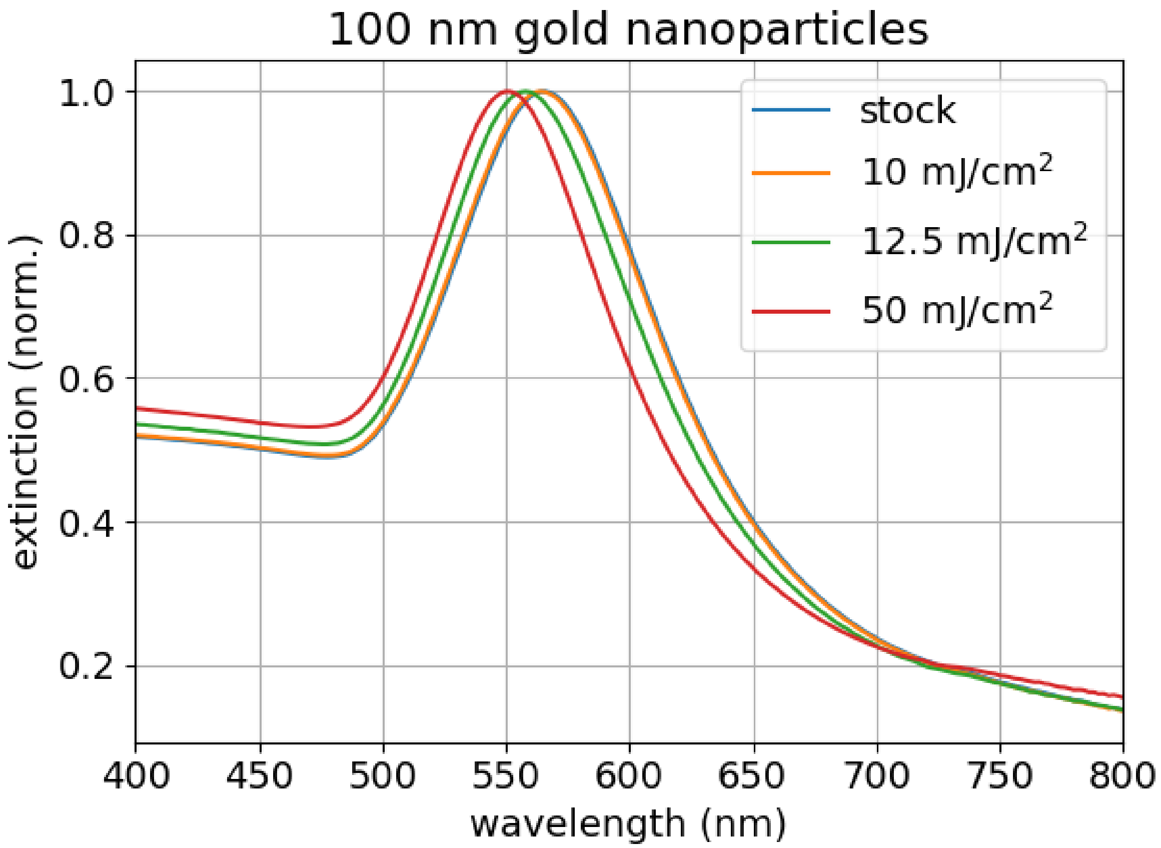

Figure S5. Representative extinction spectra of 100 nm gold nanoparticles after laser irradiation at the indicated radiant exposures.

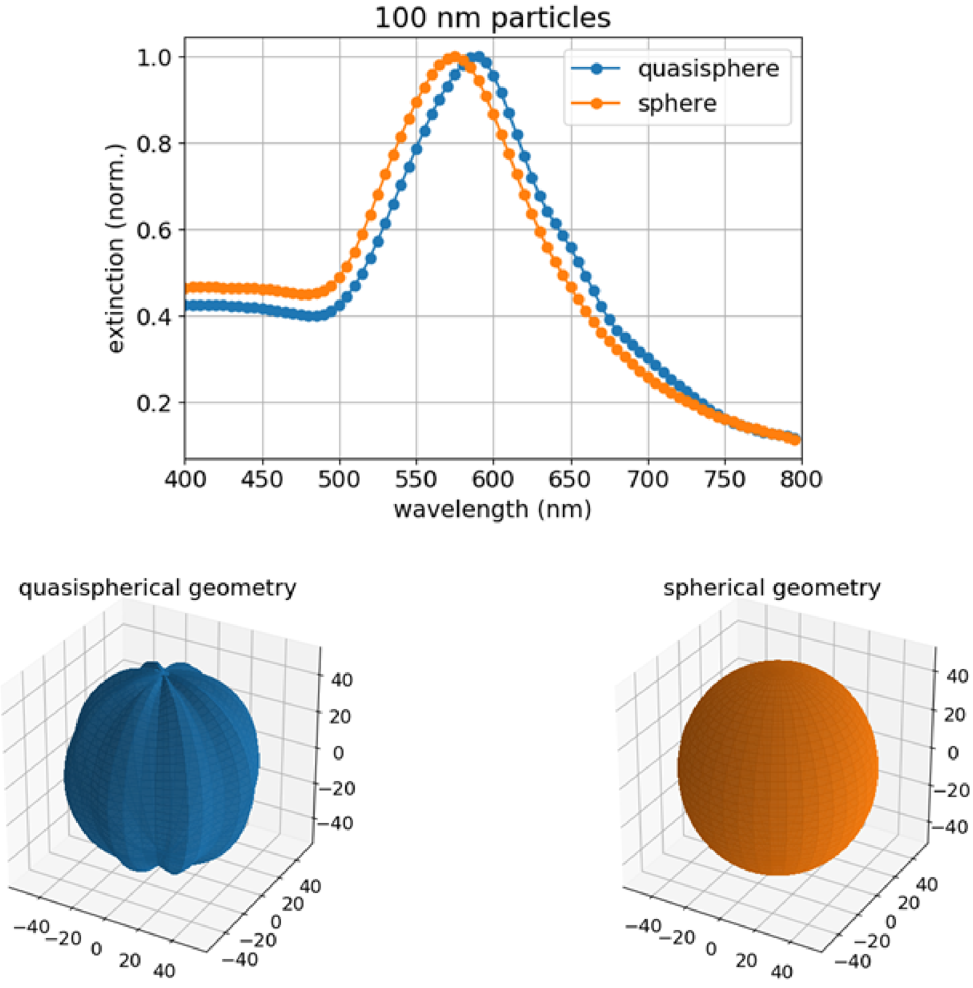

Figure S6. DDA calculated extinction spectra of 100 nm diameter spherical and quasispherical gold nanoparticle geometries. The quasispherical model employed the built in 'chebyshev' shape with Ɛ = 0.05 and n = 8.

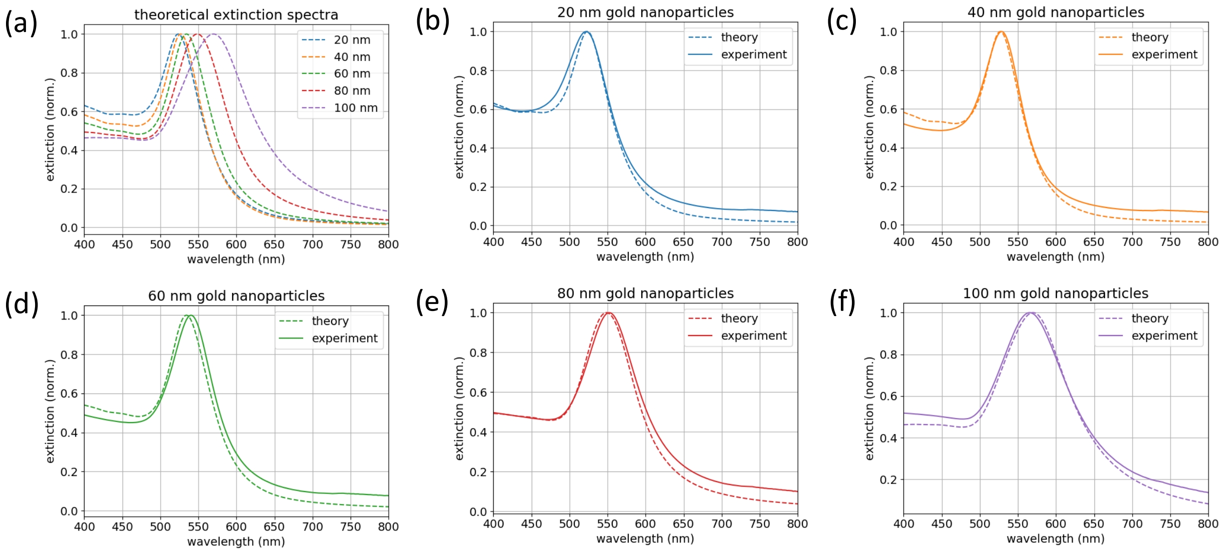

**Peak Position (nm)**	**theory**	**experiment**
**20 nm**	523.6	521.77 ± 0.04
**40 nm**	526.8	528.70 ± 0.02
**60 nm**	534.3	540.44 ± 0.02
**80 nm**	548.6	552.29 ± 0.06
**100 nm**	569.9	565.3 ± 0.1

Figure S7. (a) Theoretical extinction spectra for 20, 40, 60, 80, and 100 nm gold nanoparticles as calculated from Mie theory. The theoretical spectra were then compared to the experimentally measured spectra for the 20 (b), 40 (c), 60 (d), 80 (e), and 100 (f) nm particles.

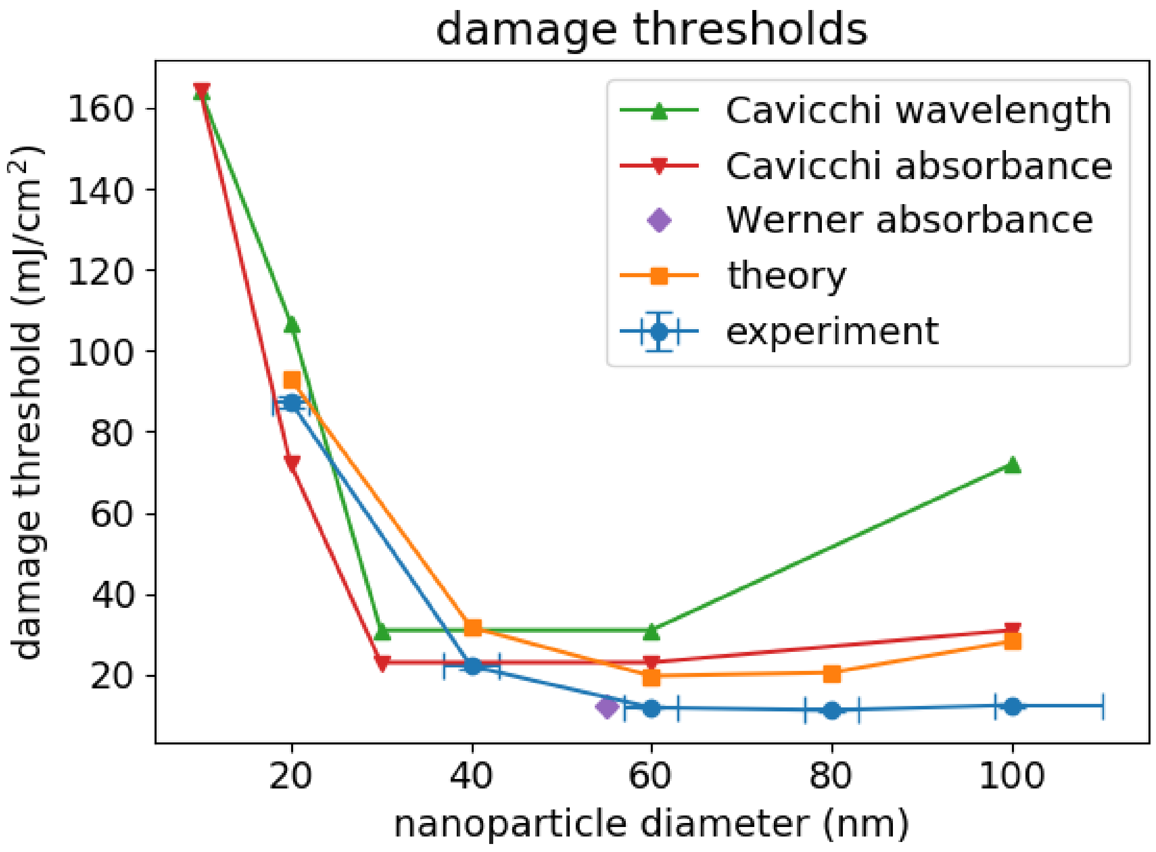

Figure S8. Comparison of the theoretical and experimental damage thresholds determined in this work with experimental thresholds found in the literature.[1,2] Data points for Cavicchi et al. were estimated from Figure 4 in their manuscript using either the midpoint of the peak wavelength shift or the first significant drop in absorbance.

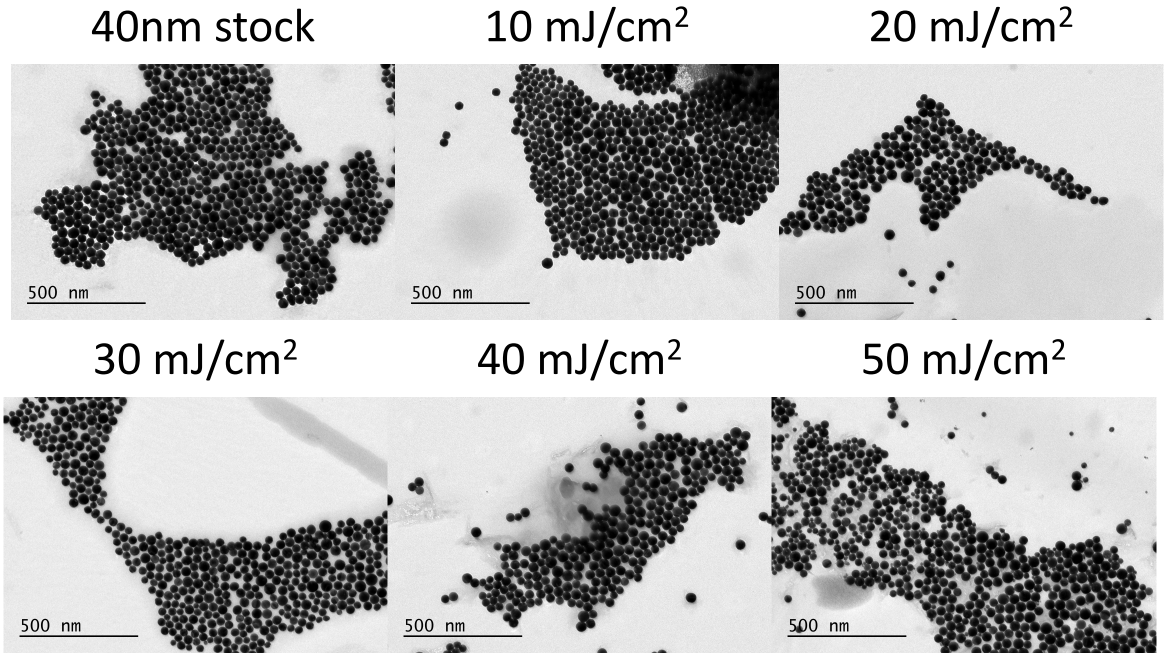

Figure S9. Low magnification TEM images of the 40 nm gold nanoparticle sample demonstrating the same reshaping and size reduction process observed in the representative images from Figure 2 with a larger number of particles.

References

1 Cavicchi, R. E., Meier, D. C., Presser, C., Prabhu, V. M. & Guha, S. Single Laser Pulse Effects on Suspended-Au-Nanoparticle Size Distributions and Morphology. *The Journal of Physical Chemistry C* **117**, 10866-10875, doi:10.1021/jp4041502 (2013).

2 Werner, D., Hashimoto, S. & Uwada, T. Remarkable Photothermal Effect of Interband Excitation on Nanosecond Laser-Induced Reshaping and Size Reduction of Pseudospherical Gold Nanoparticles in Aqueous Solution. *Langmuir* **26**, 9956-9963, doi:10.1021/la100015t (2010).
